# Collective memories and social roles: the case of the Paris terrorist attacks of 13 November 2015

**DOI:** 10.3389/fsoc.2024.1388380

**Published:** 2024-05-22

**Authors:** Jean-François Orianne, Serge Heiden, Carine Klein-Peschanski, Francis Eustache, Denis Peschanski

**Affiliations:** ^1^Social Science Research Institute (IRSS), University of Liège, Liège, Belgium; ^2^Neuropsychology and Imaging of Human Memory (NIMH) Research Unit, INSERM-EPHE-PSL-University of Caen-Normandy, Caen, France; ^3^Institute of History of Representations and Ideas in Modernities (IHRIM), École Normale Supérieure de Lyon, Lyon, France; ^4^European Center for Sociology and Political Science (CESSP), University of Paris 1 Panthéon Sorbonne-EHESS-CNRS, Paris, France

**Keywords:** collective memory, social role, mass media, November 13, textometry

## Abstract

The present study was based on empirical data collected during the first phase (2016) of Study 1000, part of the 13-November Program: a corpus of 934 individual interviews conducted 6-11 months after the events. To process this empirical material, the authors used integrated TXM software, which provides several classic textometry tools. They mainly used the lexical specificity analysis tool, which statistically measures the irregularity of the word distribution according to the parts of the corpus. They also analyzed the concordances of certain very specific lexical forms. Analysis revealed the important influence of social roles on the construction of memories and narratives of this event. Application of textometry tools highlighted lexical fields specific to the different social roles played by the interviewees in this *social drama*, and showed that it was through these specific vocabularies that they remembered and recounted this *extraordinary* story. Social roles therefore influence the formation of memories both individual and collective, by modulating the way in which individuals select what to remember and what to forget. The article opens up several interesting avenues for future analyses, mainly a longitudinal perspective (including phases 2 and 3 of Study 1000) for the study of flashbulb memories and the gender issue to fine-tune the analysis of social roles.

## Introduction

On the evening of November 13, 2015, a series of shootings and suicide attacks struck Paris and its suburbs, first at the Stade de France, then on café and restaurant terraces, and finally at the Bataclan concert hall. The official death toll was 130 (including 90 at the Bataclan) and more than 400 were hospitalized. These attacks, the deadliest in France, had a greater impact on the French population than other tragic events in France and abroad (Hoibian et al., 2020). CREDOC surveys, carried out on a representative sample of the French population, underline the singular impact of 11/13 on public opinion and highlight a mechanism of memorial condensation, reinforced recently with the trial (Hoibian et al., 2023).

This article deals with collective memories of the 13 November 2015 terrorist attacks in Paris. We analyzed 934 individual interviews conducted in 2016 for Study 1,000, part of the 13-November Program, focusing on how social roles influenced the construction of memories and narratives of this event.

According to systems theory of Luhmann (2021), social roles allow structural couplings to be established between consciousness and communication, in other words between individuals and social systems. In order to participate in a communication operation (i.e., event in social life), individuals’ systems of consciousness have to take on social roles-just as actors don *masks* when they go on stage.

We analyzed the corpus using textometry software (TXM), in order to find the topics, vocables or lexical fields that characterized the different social roles played by the interviewees in this *social drama*. According to Charles Wright Mills, “people perceive situations through specific vocabularies” (quoted by Strauss, 1992: 52). We therefore set out to highlight the specific vocabularies that participants used to remember and narrate this *unusual* situation.

After defining the main theoretical constructs (i.e., concepts of collective memory and social role) we used to refine our research problem, we describe the Study 1,000 corpus and our research methodology. We then present and discuss our main findings in the form of a *cast list*.

## The concept of collective memory

The concept of *collective memory* was introduced in the 1920 and 1930s by Maurice Halbwachs, a French sociologist and direct heir to the sociology of Émile Durkheim. Halbwachs based his sociological study of memory on an original intuition that “we never remember alone”: “in this sense, there is a collective memory and social frameworks of memory, and it is because our individual thinking takes place within these frameworks and contributes to this memory that it is capable of remembering” (Halbwachs, 1925: Foreword). His study of collective memory among musicians underlined the importance of *social frameworks of memory*, without which neither individual nor collective musical memories would be formed (Halbwachs, 1939). These social frameworks of memory should not be confused with musicians’ individual and collective memories. If people remember more or less the same facts when they share a common experience, it is because a social system that is external to them affords them this possibility (Halbwachs, 1947, 1950).

Since the 1980s, a less precise notion of collective memory has been used in the social sciences to study phenomena as varied as shared memories and narratives (Knapp, 1989; Smith, 2004), commemorations (Schwartz, 1982), myths and cultural scripts (Green, 2004), and broader culture (Coser, 1992). In the field of cognitive neuroscience, enthusiasm for Halbwachs’ work has inspired a veritable *social turn* (Coman et al., 2009). Be it a metaphor or a reality, the concept of collective memory lies at the heart of the most heated debates (Laikhuram, 2022). It is used in empirical studies adopting two complementary approaches: (1) the top-down approach, which takes social frameworks (social representations, narrative models, cultural patterns, etc.) as its starting point; and (2) the bottom-up approach, which explores (in the laboratory) how individuals share memories (Hirst and Manier, 2008), mainly by studying dyadic exchanges, the idea being that what is observed at this local level shapes what emerges at a more general level (Hirst et al., 2018). Our analysis clearly espoused a top-down approach: without any prior consultation or discussion, interviewees recounted *their* November 13, and their accounts were then analyzed to determine what they had retained overall and how they had filtered what to remember and what to forget. From a sociological point of view, we regarded the collective memories of the November 13 attacks as *collected memories* (Olick, 1999), reconstructed by a research team from individual accounts of the attacks.

Recent work on collective memory has provided us with four essential notions for characterizing our subject and constructing our research problem: (1) *collective memory* refers to the memories of individuals as members of a group or community or as participants in an interaction (Roediger, 2021; Wertsch and Jäggi, 2022); (2) collective memories are formed through processes of identity construction (Booth, 2008; Bachleitner, 2022; Fischer and O'Mara, 2022); (3) in terms of selecting what to remember and what to forget, the essential function of collective memory is to forget (Vinitzky-Seroussi and Teeger, 2010; Kevers et al., 2016; Hirst and Coman, 2018); and (4) unlike history, which separates the past from the present and the future, collective memory connects all these strands (Crane, 1997; Assmann, 2008), in that it operates in the present as a continuous rewriting of the past for future use (Schwartz, 1997; Adams and Edy, 2021; Peschanski, 2021; Bachleitner, 2022). While *memory studies* since Halbwachs have made it possible to identify and study multiple *social frameworks* of memory, such as schemas, scripts, and symbols, the issue of social roles is curiously absent from work on collective memory (Orianne, 2023; Orianne and Eustache, 2023). However, we feel that it is essential for understanding how individual systems of consciousness access and contribute to the communicative operations that make up society and its multiple social systems.

## Social roles

The concept of *social role* is generally used to denote a system of normative constraints and associated rights: “the role therefore defines a zone of obligations and constraints that correlates with a zone of conditional autonomy” (Boudon and Bourricaud, 1982, 505). This concept, central to sociology, has its roots in the development of the discipline in the United States. The first traces of it can be found in the pioneering work of George Herbert Mead, particularly in his sociological theory of *mind* and s*elf*, based on a theory of social roles:

Thinking involves not only communication, in the sense of the vocalization of birds, but also the production in the individual himself of the response he provokes in others, the assumption of the role of others and the tendency to act like others. We participate in the process that the other individual is engaged in and guide our action by reference to that participation (Mead, 2006, 157).

The metaphors of *play* and *game* can help to explain the dialectics of the *Me* and the *I* that constitute the *Self*. For the *Ego*, the formation of the self involves the diversions via *Alter*, and the development of the ability to take on the role of the other (i.e., what Piaget called *decentering*). The concept of role-taking is central here: people can put themselves in the role of the other and find a standpoint from which to observe themselves (Luhmann, 2001, 76).

Drawing on Mead’s theoretical insights, Talcott Parsons defined the concept of *social role* as a system of anticipations (instrumental, expressive, and moral) linking the person performing the role to those for whom it is performed (Parsons, 2004, 67–72). According to Parsons, roles encompass the fundamental areas of interpenetration between the social system and the individual’s personality. Parsons also emphasized the notion of *role pluralism* (i.e., people’s membership of several different communities is an essential characteristic of human societies; Parsons, 1973a), especially so in modern societies (Parsons, 1973b, 13).^1^ In this context, the separation of roles is essential. As Erving Goffman noted

each individual plays more than one role, but the “segregation of audiences” saves him from contradictions, since those before whom he plays one of his roles are not usually those before whom he plays another, which allows him to assume several characters without discrediting any of them (Goffman, 1974, 96–97).

Role contradictions and conflicts have been an important research topic, especially within the Chicago School. The interactionist perspective offers an interesting counterpoint to Parsons’ functionalist approach, which emphasizes the stabilizing and unifying function of social roles: on the one hand, it questions the origin and formation of social roles; on the other, it questions role-taking (and its infinite psychological nuances), using a theatrical metaphor.^2^ Another essential contribution of the interactionist approach is the concept of *expressive distance from the role*, which refers to the ability to distance oneself from the official character and imposed universe, with these secondary adaptations providing the individual with “the means of departing from the role and the character which the institution assigns to him quite naturally” (Goffman, 1968, 245). The theatrical metaphor naturally invokes the concept of the *mask* (Strauss, 1992, 60) when thinking about the multiple role-playing in which human actors may engage.

In the theory of social systems of Luhmann (2021), social roles fulfill an essential function, by enabling forms of structural coupling between individual systems of consciousness and systems of communication (i.e., society). Based on these theoretical foundations, we formulated our research hypothesis, whereby social roles influence the formation of memories (individual and collective), and modulate the selection of what to remember and what to forget. If memory is closely linked to the *self* (Conway, 2005), it is because social roles, through which identities are formed, frame and guide the cognitive operations of individual systems of consciousness. As we saw earlier, to participate in a communication operation or a social event, individual systems of consciousness take on social roles, just as actors don a *mask* to go on stage. It is through the prism of a role that consciousness can connect with society, functional systems (as a client, beneficiary, student, parent, etc.), organizations (as a member), and systems of interaction (as a participant), and can perform the operation of selecting what to remember (both encoding and retrieval) and what to forget.

## Materials and methods

### Corpus

The present study was based on empirical data collected during the first phase (2016) of Study 1,000, part of the 13-November Program. For the purpose of this research on memories of the traumatic terrorist attacks that took place in Paris and Saint-Denis on 13 November 2015, almost a 1,000 people, divided into four circles, have already been interviewed three times (2016, 2018, and 2021) about their recollections of these events.^3^ The witnesses all agreed to take part on a voluntary basis (see Nattiez et al., 2020). The first circle is made up of people who were exposed (survivors and their relatives, professional responders, bereaved families, and direct witnesses). The second circle is made up of people (other than witnesses) who were living or working in the neighborhood at the time. The third circle is made up of people from other districts of Paris or municipalities in the Paris metropolitan area, and the fourth circle is made up of people from other French cities (Caen, Metz, and Montpellier). 13-November is a long-term research program (2016–2028) that is resolutely transdisciplinary, bringing together the humanities and social sciences (the focus of Study 1,000), as well as the life sciences and engineering (Eustache and Peschanski, 2022).

The construction of the sample was based on the hypothesis that memories of the attacks vary between these concentric circles [i.e., interviewee’s geographical position (center/periphery)], as well as within each circle according to witness category. This hypothesis is borrowed from the work of Hirst and colleagues on the long-term memory for the terrorist attack of the September 11 in New York (Hirst et al., 2009, 2015).^4^ Our aim here was to test this hypothesis by rephrasing it in sociological terms: *social role*, as reflected in the words used by each person, and *distance* from the event both influence the formation of individual and collective memories. On the question of what these 934 individual testimonies have in common, we hypothesize that the social role makes it possible to explain and interpret the most significant differences, and thus to make the collective memory of November 13 appear as the contingent result of sorting operations (between forgetting and remembering) common to different social groups or types of participants.

The present study focused on data collected in Phase 1 (i.e., 934 interviews conducted 6–11 months after the events (Table 1);^5^ these are the only data currently available.^6^ All interviews were conducted between May 9 and October 31, 2016 (before the first commemoration); the four circles in parallel. The corpus for Phase 1 represents a total of 1,431 h of recording, or an average of 1 h 30 per interview; for Circle 1, the average is 2 h 30. The interviews were recorded, filmed either in the studios of the National Audiovisual Institute (INA) or by mobile teams from the Defense Department’s *communication and audiovisual* production unit ECPAD), and transcribed in full. Interviewees also completed a sociodemographic questionnaire and a questionnaire about their recollections of the events off camera. The filmed interviews came in two parts: a semistructured interview based on three main questions (telling *your* story of 11/13, telling *the* story of 11/13, interpreting 11/13 in terms of causes and consequences); and a structured emotional memory questionnaire. We considered all the semistructured interviews. The words of the interviewers were not counted-only the words of the witnesses.

**Table 1:** Study 1,000 (Phase 1): breakdown of testimonies (*N* = 934) according to Circle.

Circle	*N*	Category	% Woman	% 18–39 y.o.
Circle 1: Attacks	359	Survivors (112), professional responders (138), bereaved (42), witnesses (42), families & loved ones (25)	49%	57%
Circle 2: Affected neighborhoods	144	Residents (103), users (41)	73%	59%
Circle 3: Ile-de-France	147		65%	61%
Circle 4: Other French cities	283	Caen (117), Metz (76), Montpellier (84), Other (6)	61%	42%
*Not coded*	*1*		*0%*	*100%*
**Total**	**934**		**59%**	**53%**

### Methodology

To process this empirical material, we used integrated TXM software (Heiden et al., 2010), which provides several classic textometry tools (Peschanski, 1988; Lebart et al., 2019). We mainly used the lexical specificity analysis tool, which statistically measures the irregularity of the word distribution according to the parts of the corpus (here, according to social roles): in other words, we listed the words that occurred abnormally (in)frequently for each social role, together with a statistical index reflecting their degree of over-or under-representation.^7^ We also analyzed the concordances of certain very specific lexical forms. This allowed us to look at the contexts in which words were used in a synthetic and methodical way, in order to understand the precise meanings(s) that were given to them and by whom (each word acted as a pivot and had neighboring words on both sides). Concordance analysis made it possible to identify and select the extracts from the testimonies in which the most specific lexical forms appeared.

Returning to the way in which the material was collected, within a very specific interactional framework (a *staged event* in the Chicago interactionist sense), people were asked to *play the game* of testifying for a scientific research program. After a short and unsystematic make-up session, a few technical tests and a short briefing, participants were each asked to tell the story of *their* 11/13 and then *the* 11/13. These narratives were followed by their interpretations (i.e., meanings) in terms of causes and consequences. This interview situation reinforced the logic of self-presentation, compared with an interview conducted directly in the field (Antichan, 2017), and encouraged certain behaviors. We can assume that participants each felt the need to demonstrate the legitimacy of their testimony, its coherence, the accuracy of the factual details, the authenticity of their feelings, the uniqueness of their perceived meanings, and so on.^8^ The social conditions under which these testimonies were produced led us to consider the social role of the *subject of study* in our analysis of the construction of the narratives, as well as the singularity of the witnesses’ sociodemographic characteristics. The sample was by no means representative of the French population (Cayre, 2020), as there was an over-representation of women (as is often the case in corpora based on voluntary participation), managers and higher intellectual professions, and university graduates (up to 92% in Circle 3, compared with only 41% in French reference population). It was, however, close to the categories that were directly affected, particularly in Circle 1.

Despite a fascinating and rich literature on flashbulb memories (see Luminet and Curci, 2017), this approach was not used in this study for three main reasons. Firstly, the concept is only relevant to part of our sample: if flashbulb memory corresponds to the detailed recollection of the circumstances in which an individual is informed, by an external source, of an unexpected, emotionally charged and socially important event (Brown and Kulik, 1977), then this concept would only apply to circles 3 and 4 of our corpus, to people who were informed by an external source; everyone else experienced the event live.^9^ Secondly, the flashbulb memory approach implies a longitudinal dimension in the analysis (e.g., Dégeilh et al., 2021), which is currently impossible as only data from phase 1 are available. Finally, our aim here is not to study the recollection of the circumstances in which individuals are informed of the event: our aim is to study the memory narratives of this event, what people experienced based on their position in the social configuration of the event, the role they played in this drama.

We worked on the assumption that the social roles could be studied from a lexical point of view, with each social role having a specific vocabulary. The social roles that received the greatest media coverage were those of the survivor and the professional. Then there were the bereaved, and the close friends and relatives of the survivors. These individuals belonged to the private space, the realm of personal relationships. Direct witnesses and local people belonged to the realm of impersonal (even anonymous) relationships that characterize any public space (here, the street). Finally, remote spectators belonged to two categories: the center or the periphery. We selected the following categories or subcorpuses for our study (Table 2). We then looked at the lexical forms that characterized these social roles in the corpus and how they structured the narratives. To this end, we calculated the specificity of the division of the corpus into the eight social roles, and for each role we collected and interpreted the words with the highest specificity indices.^10^

**Table 2:** Study 1,000 (Phase 1): Categories selected for analysis.

Social role	Circle	Number of testimonies	Subcorpus length (in words)	Words per witness
Survivors	1	112	2.663.835	23.784
Professional responders	1	138	2.594.003	18.797
Bereaved families	1	42	1.025.903	24.426
Close friends/relatives of survivors	1	25	529.167	21.167
Direct witnesses	1	42	887.568	21.133
Local people	2	144	2.276.621	15.810
Remote spectators: center	3	147	2.186.649	14.875
Remote spectators: periphery	4	283	1.735.278	6.132

## Survivors

The survivors’ accounts were among the longest (along with those of the bereaved). They were written in the first-person singular: *I* (specific index: 1,000) and *me* (specificity index: 1,000).^11^ These accounts were characterized by a direct, descriptive style that was rich in content and relatively familiar. Most of the people had already given evidence (to police, journalists, charities, lawyers, etc.). Of the 112 survivors in the sample, 79 had been in the Bataclan, 17 on the café terraces,^12^ 12 in the Stade de France, and four in the vicinity of one of these places. It should also be noted that 53% of the survivors’ testimonies were given by women.

The textometric analysis revealed three sets of lexical forms that structured the survivors’ narratives, taking the form of a three-stage *narrative template* (Wertsch, 2008), a temporal succession of specific social roles: spectator/consumer, then victim, then survivor (Table 3).^13^ As Parsons noted, “a role is a sector in the system of orientations of individuals, organized around anticipations relating to a particular context of interaction, or integrated into a particular set of evaluative criteria governing the interaction of one or more subjects” (Parsons, 1963, 54).

**Table 3:** Spectator/victim/survivor: specificity indices.

Social role	Category	Lexical form (specificity index)
Spectator at Bataclan concert	Musical event	Concert (224), Eagles (51), Death (48), Metal (42), band (41), song (20), rock (19), drums (17), music (14)
	Bataclan	Pit (174), hall (108), door (66), exit (64), stage (61), staircase (60), balcony (50), bar (36), toilet (31), dressing room (30), ceiling (20)
Victim	Attacks	Shoot (146), man (143), bullet (140), shot (77), floor (65), ground (64), wound (49), reload (34), three (19), gunpowder (16), shooter (15)
	Action verbs	To go out (121), to run (61), to lie down (53), to shout (52), to walk (43), to fall (38), to die (36), to crawl (36), to help (31), to pity (30), to lift (30), to wait (28), to scream (25), to breathe (26), to shout (17), to calm (17), to stress (17), to trample (14)
	Perceptions	Noise (65), firecracker (50), light (41), smell (40), scream (31)
	Body parts	Blood (67), arm (56), leg (55), hand (50), head (42), thigh (22), shoulder (16)
	Clothing	Jacket (20), T-shirt (20)
Survivor	Role name	Alive (35), survivor (34), survival (20), lucky (14)
	Medical vocabulary	Psychologist (55), hospital (44), psychiatrist (32), therapy (31)
	Legal vocabulary	Affidavit (70), lawyer (29), complaint (18)
	Means of transport/communication	Taxi (29), telephone (24)

The roles of spectator in the Bataclan (or Stade de France) and consumer on a café terrace were associated in most of the testimonies with other social roles (family, private, and professional), such as attending a concert with friends, having a drink with colleagues, or watching the match with other supporters. A first set of very specific lexical forms in this subcorpus characterized the role of spectator at the Bataclan, during the Eagles of Death Metal concert (Table 3). It should be noted that from the perspective of *collected memory* based on individual memories, the over-representation of people present at the Bataclan (70.5%) inevitably had the effect of crushing (or obscuring) the other testimonies.

Through the prism of this ordinary social role of spectator, something extraordinary suddenly emerged (moment when everything changed): *moment* (87), *instant* (15). Many of the accounts describe the impossibility of bringing the strange into the familiar, the unknown into the known. The following excerpts illustrate the many attempts made by witnesses who had been spectators at the Bataclan to interpret the situation that emerged (Table 4): *joke*, *firecracker*, *hoax*, *joker*, *technical problem*, *staged effect*, and so on. In the end, it was their repeated failures that led to a change of role from spectator to victim (undergoing the attack).

**Table 4:** Role of spectator at bataclan concert: extracts from testimonies.

And then, after a while, I actually hear something like firecrackers…something…at this point…so I had the mixing desk right behind me, I am a musician: it sounds like a cable being unplugged and something banging in the speakers. I turn round to the console, I try to look at it and, at the same time, I say to myself: “This is absurd! The sound is coming from the front, not the back! If the sound was crackling in the mixer, it would be crackling at the front, not at the back! So it’s something else” (*PAR0147-Bataclan*).
When the bombs went off, well they went off, and we did not understand what it was. Because we were jumping, dancing, we were happy, well, it was really a moment of pure happiness, so to switch to the other extreme was really…well, it was complicated. And it took me a long time to realize what had happened and to realize it consciously, to say to myself: “What’s going on…there are actually people shooting guns.” I was always trying to extrapolate…“It’s panic, it’s firecrackers.” (…) And I…I looked over to see what was happening and I saw… I saw a man standing at the back. And the spectators who were lying in the pit, I did not see any blood, I did not…And my brain refused to see what it was, I said to myself: “Oh, it’s beautiful, it looks like wheat lying down.” But I could not see, I did not see any weapons, I think that, well I saw him standing at the bottom of the pit, I think that…he had it. I saw it, but I did not… I did not register it. And…so we crawled out along the balcony and…we got up and went through the door, we found ourselves in a stairwell (*PAR0331-Bataclan*).
And the first thing I heard was noise, quite loud, quite clear and intense noise. Excuse me, I had two initial reflexes: the first was to say to myself stupidly: “the drummer’s hitting it hard.” Because the noise was covering, well I quickly realized that it was covering the noise of the snare drum, but as a musician the first reflex I had was to say to myself, that is strange, he is hitting it hard when he is playing superbly, there is something wrong, I can hear the noise of the snare drum, but they are not in rhythm, and they are much too loud. And then the second, the second thought I had, but it was immediate, it happened in a split second, the second thought I had was: “and I turn toward the source of the noise, which is the entrance to the room, and then, and then it goes on and on, and I start to hear people shouting and saying it’s firecrackers, it’s firecrackers, it’s a joke, it’s a bad joke and so on. And stupidly, in the first moments, when I thought it was actually a bad joke and I heard people shouting things in Arabic, I said to myself, this is really the worst joke you can make at a concert, it’s really completely stupid. (…) It was only when I saw the drummer leave that I realized it wasn’t a problem with the drums and it wasn’t a problem with the sound system, so again it all got mixed up very quickly in my head” (*PAR0274—Bataclan*).

A second set of lexical forms was directly related to the role of the victim (Table 3). These were used to name the attackers (*three guys*) and to describe the attack and the victim’s posture (*lie down*, *fall*, *crawl*, *pity*, etc.), with many factual details and the perceptions associated with them (sound of gunshots, metallic sound of Kalashnikovs, screams, strong smell of gunpowder and blood,^14^ flashes of light from the explosions, sight of blood flowing copiously,^15^ being slipped on, etc.). Body parts and certain items of clothing (covered in gunpowder, blood, etc.) formed a specific vocabulary for describing the people present and the participants themselves as victims. The participants said a lot about these bodies, the organs and limbs that people tried not to trample on as they fled, that they did not look at too closely as they left, that they could not forget, and so on.

Taking on the role of victim often coincides with a change in state of consciousness, which is what the interviewees described. In psychopathology, this is known as *dissociation*. Brought on by extreme anxiety, it is mainly characterized by a change in temporal reference points: the normal experience of time passing in a linear and continuous way suddenly gives way to a succession of snapshots, moments that never end, endless waiting, and so on. There is also a change in the way the world is viewed.

Some of the interviewees related highly unusual subjective experiences of awareness (comparable to an awakening) after a state of anesthetic anguish or shock, and described in great detail the cognitive and cerebral mechanisms that made it possible to adopt a survival strategy (i.e., becoming a survivor). In the first excerpt, the aim was to adopt the attitude (role) of a war reporter (accepting death) in order to be able to analyze the situation and act appropriately. In the second extract, the aim was to take inspiration from video war games (*Call of Duty*) in order to change posture and take on the role of survivor. In both cases, taking on a role allowed the system of individual consciousness to participate in the interaction process, generate new interpretations of the situation, anticipate the behavior of others (and of self), and guide the individual’s action according to this participation (Table 5).

**Table 5:** Role of victim: extracts from testimonies.

As if, in order not to die, all you had to do was not want to. I am doing a kind of self-coaching, like this: want it very much, hold on…that is it. So I concentrate on not moving, on breathing without my back…without it showing. And then I tell myself that if I survive, I will have to testify. I am a journalist, so I start to think of myself as a war reporter, which is not my job at all, because I work in film, so it is really nothing to do with that. I am not at all cut out for this kind of…but whatever. I tell myself that I have to analyze everything that happens, that I have to record the smallest sound, the smallest gesture, the smallest vibration. And the floor is wood, it transmits sound, so I…it is like being a Sioux with my ear pressed against the floor. I am trying to see if there are people walking around, if there are still people alive around us who are moving or not or…well, I am trying to understand what is going on. (…) At this stage, I am holding on to a lot of things, because you have to…because, to survive, you have to…there are two things. The first is something that…somehow makes your brain twitch, but to have a chance of surviving, you have to stay calm, stay calm, you have to calmly accept that you are going to die. It is a crazy contradiction but…but that is what I ended up telling myself. So at some point I had to consider that my son would indeed be an orphan (*PAR0147-Bataclan*).
I had this moment when I let go completely. (…) There was this moment when I totally let go and I remember…I remember looking at Thomas and saying to myself: “You’re not getting out of this place. I was resigned. And then, er…there was…Right after that there was a moment of awareness, a survival instinct maybe.” And I really like video games. I know that might sound irrelevant right now, but you’ll see, it makes sense. Because I used to work, I worked for 10 years for a small TV station where I was a journalist and I talked about video games from morning till night. And that moment when my survival instinct took over, when I raised my head. I was…I analyzed the situation as I would have analyzed it in a war game, in a *Call of Duty*, *Battlefield* or whatever. It is totally, totally absurd what I am saying, but at the time I lifted my head up and said to myself: “Here, try to analyze the situation, try to really understand what’s happening. See where you can get out, see who’s doing what and see who’s got what weapon in their hands.” And so I saw that the guy on the left, dressed in black, was reloading his gun. His back was turned. And I saw that the guy on the right was shouting at people, but his back was also turned. It all happened in a split second. I looked at Thomas. I was petrified, I was really I, I, I, I could not make a sound, but I looked at him, I nodded to him to show him the door and make him understand that I was going to get up. (…) So I looked at him, I nodded, and I looked at the young man who was, who was lying on top of my legs. I pulled a little on my legs and he understood that I wanted to get up. He moved slightly to the side. I got up and lost a shoe. And I ran away (*PAR0274-Bataclan*).

A third set of lexical forms referred specifically to the role of survivor (Table 3). In addition to naming the role, survivors used medical and legal vocabulary to describe the steps and obligations arising from this social role (filing a complaint, starting therapy, and getting better). Taxis (29) seemed to be the main means of transport used by survivors to get home. The telephone (24) was the main means of communication for notifying relatives and the emergency services.

Several interviewees described identity tensions and contradictions between the role of victim and that of survivor (see also Dénouveaux and Garapon, 2019). For some, it was important to relinquish the social role of victim, “in order to turn the page” (PAR0074). For others, it was inconceivable that they should be viewed as victims (PAR0036). Some spoke of a feeling of illegitimacy regarding the role of victim, especially when they had not been physically injured, as shown in the two extracts below (Table 6). The difficulty of seeing themselves as victims seems to have been a form of *role denial*. The question of *self* is central to the production of narratives.

**Table 6:** Role of survivor: extracts from testimonies.

But it took me a long time to see myself as a victim or collateral damage of the events. And even today, I sometimes ask myself: “But…where’s the trauma? But the fact of doing this movement, I realized…well, at that point, I kind of accepted the idea that despite everything, despite everything, I was an indirect victim of the events, but a victim nonetheless, because, because I needed to do something to myself to get back there, because…because it brought tears, because…because I could not deal with it at all (*PAR0107*).
But as a victim of 13 November nothing happened to me physically. So I do not feel like a victim when I see everything that happened around me and all the people that were injured and all the lives that were turned upside down, I feel, well I do not think I am on the same scale of seriousness in fact as these people, so I cannot consider myself a victim (*PAR0332*).

The very specific vocabulary of the survivors contrasted sharply with that of the professionals. As we will see, these two lexical fields underpinned contrasting memories of the event.

## Professional responders

There were four types of professionals in this subcorpus: the forces of law and order (police and army), health professionals, first aiders, and politicians and civil servants (Table 7). The forces of law and order were overrepresented (44%), as their social role received the most media coverage after that of survivors. It should be noted that 75% of the testimonies of professional responders were from men. This is the only category of testimony in which women were underrepresented.

**Table 7:** Professional responders (*n* = 138): categories and numbers.

Category	Professional role (*n*)
Law enforcement (*n* = 61)	Search, Assistance, Intervention, Deterrence (RAID) unit/Investigation and Intervention Brigade (BRI) police officer (33), technical police officer (investigator) (3), dog handler (explosives) (2), bomb disposal expert (2), driver (police) (1), brigade/team/department commander (3), police commander (2), police major (1), police director (1), BRI chief (1), BRI officer (1), police commissioner (11)
Healthcare professionals (*n* = 21)	Nurse (5), general practitioner (1), emergency physician (2), BRI physician (1), medical student (1), medical assistant (1), head of hospital department (1), psychologist (6), psychiatrist (3)
First aiders (*n* = 21)	Red Cross (9), first aid (civil protection) (3), fire brigade (9)
Politicians and civil servants (*n* = 24)	Elected official or city employee (Paris, 10 and 11th districts) (17), deputy (2), Élysée Palace cabinet member (2), President of the Republic, Secretary of State for Defense (Veterans), Minister of the Interior
Other (*n* = 11)	Lawyer (assistance to victims) (3), city street cleaning department (2), journalist (2), security guard (Stade de France), (2); school principal (1)

In contrast to the first-person singular narratives of the survivors, the narratives of the professional responders were characterized by the use of the impersonal third-person singular *one* and the first-person plural *we*. These were the pronouns with the highest specificity indices. The pronoun *us* also had a high specificity index (65). While the impersonal *one* may partly have reflected the impersonal nature of the function (i.e., professional role), *we* referred to the work group (department, team, brigade, etc.). It should be noted that in some cases, the French pronoun “*on*” (*one*) may have been used semantically as a colloquial equivalent of “*nous*” (*w*e), thereby reinforcing the interviewees’ membership of a team or collective.

After these pronouns, the most frequently used lexical forms in this subcorpus, with the maximum specificity indices, were *victim*, *colleague*, *service*, *intervention*, *vehicle* (1,000). Here, the professional role was central to the description of events. These five lexical forms delineated the semantic field within which the memories were reconstructed: the objective (saving the victims), the work group (department and colleagues), the material resources and work tools (symbolized by the vehicle), and the intervention itself (see Table 8).

**Table 8:** Professional terminology as a discursive universe: specificity indices.

Category	Lexical forms (specificity indices)
Services present	Investigation and Intervention Brigade (BRI) (245), police (203), Search, Assistance, Intervention, Deterrence (RAID) unit (116), fire brigade (128), bomb disposal (108), Red Cross (93), first aid (90), police officer (78), judiciary (77), dog (76), rescue (76), medical and psychological emergency units (CUMP) (68), emergency medical services (SAMU) (64), psychologist (52), medical (49), demining (45), doctor (44), responder (41)
Ranks	Chief (225), commissioner (155), constable (98), officer (97), director (79), deputy (54), crew member (36), officer (35), investigator (35), captain (35), major (31)
Work group	Column (222), team (211), manpower (159), barracks (128), brigade (100), unit (100), police station (98), operational (center or support) (94), crew (92), reinforcement (62), unit (crisis or emergency) (58), emergency (53), zone (51)
Orders	Mission (146), charge (130), assault (120), waves (radio) (114), debriefing (92), device (87), perimeter (79), command (65), hierarchy (55), staff (47), direction (46), securing (45), progress (42), procedure (39)
Politicians and civil servants	Civil servant (145), city council (144), mayor (134), district (115), prefect (99), president (67), minister (65), prefecture (52), authority (39)
Work tools	Jacket (127), material (123), gear (114), shield (88), equipment (75), weapon (74), explosive (69), protection (47), armament (33), bulletproof (32), cartridge (27), helmet (27), ammunition (22), grenade (21)
Victims	Person (60), body (50), injured (47), wound (31), corpse (29)
Action verbs	Intervene (174), manage (168), equip (107), evacuate (88), advance (74), secure (72), train (54), organize (53), prepare (43), neutralize (43), fight back (20), direct (20)
Targets	Terrorist (59), suicide bomber (33)
Location	Bataclan (35), stadium (35)

The lexical forms most frequently used by the professionals to describe the victims were *person* (60), *body* (50) (bodies were identified, on the ground, in pieces, piled up, lying down, inert, everywhere, tangled, piled up, and bleeding), *injured* (47), *wound* (31), and *corpse* (29). The verbs most frequently used by these professionals to describe their response were *intervene* (174), *manage* (168), *equip* (107), *evacuate* (88), *advance* (74), *secure* (72), *train* (54), *organize* (53), *prepare* (43), *neutralize* (43), *fight back* (20), and *direct* (20). Table 8 shows the importance of this discursive universe characterizing the exercise of a profession.

Professional jargon was omnipresent in these accounts: it was the specific vocabulary through which the systems of consciousness perceived the situation, made sense of it, and selected what to remember and what to forget, as is clearly indicated by certain lexical forms with particularly high specificity indices, such as *professional* (84), *training* (61), *experience* (51), *field* (50), and *work* (47), and *trade* (47). Collective memories and professional identities seemed to be inextricably linked (see also Chevandier, 2022). Two other categories of terms were used specifically within this subcorpus: those referring to the perpetrators, such as *terrorist* (59) and *suicide bomber* (33), and those referring to the locations of the attacks, such as *Bataclan* (35) and *stadium* (35).

The stories told by the professionals also followed a three-stage *narrative template*: (a) taking on the professional role; (b) moving from the ordinary role to the extraordinary role; and (c) returning from the mission (back to the family, back to work, and back to a normal life).

They generally began by describing how they took on their professional role (except for those who were already on duty): following an urgent call, most of them had to leave a family role or step out of the private space to take up their duties, put a uniform on, and go out into the field.

Unlike the survivors, there was no subsequent need to change roles, as the professional role allowed the extraordinary to enter into the ordinary, and the unknown into the known. Some testimonials emphasized the importance of training, simulations, and previous professional experience (Table 9).

**Table 9:** Role of professional responder: extracts from testimonies.

But it is true that I was, I was glad I had military training before joining the police, because we are not trained at all to intervene in this kind of situation. In this case, it really was a war zone in the middle of Paris. When I evacuated the victims, it was the evacuation of people with gunshot wounds that I had learnt, that I had learnt in the army. When I took cover, I opened up angles and made progress, I really went into automatic mode, I did what I had learnt as a soldier (*PAR0752*).
At the time…I said to myself, well, to get people to understand what it was like for me, this could have been a war zone. To understand a little, to try and make people understand, it is a neighborhood where you hear cries for help, where there is blood everywhere…that is it. You always have to be on the lookout, to know if someone’s coming, if…That is it, a war zone (*PAR0756*).
When I talk to my RAID colleagues, they tell me it is a scene…It is a scene they are used to seeing in Kabul, not in the middle of Paris in 2015. It is…we are in a war context (*PAR0807*).
And we came to the café La Bonne Bière, which is at the corner of Fontaine-au-Roi and Faubourg du Temple. And that is when we saw the first victims, people lying on the ground with…terrible wounds! War wounds, that is the word that came back very often from everybody…after…I mean after a debriefing (*PAR0741*).
We went to the entrance of the Bataclan and started to evacuate as many victims as possible from the lobby that led to the orchestra pit. It was…Let us just say that at that point it was quite. It was a war zone at the entrance to the Bataclan, there was blood everywhere, there was blood mixed with pieces of broken glass. There were colleagues who, when they were evacuating the victims, fell straight into the pool of blood because it was just everywhere (*PAR0752*).

**Figure 1:**
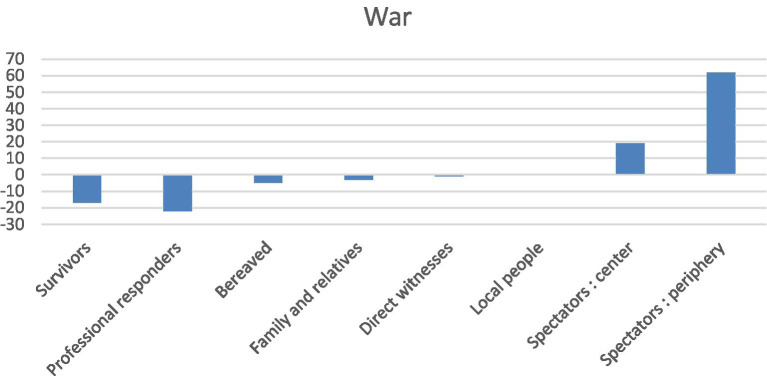
Specificity index for war according to social role.

The war metaphor was used by some to express the extraordinary nature of the intervention. As soon as the very first TV interviews had taken place, this powerful image was taken up by the mass media. On the evening of the attacks, the French President also declared “We are at war.” It should be noted in this regard that the term *war* was certainly not specific to the accounts of professional responders-quite the opposite (negative specificity index: −22), as it was a lexical specificity of remote spectators, as shown below (Figure 1). By contrast, the expressions *war zone* (5), *weapon(s) of war* and *battlefield medicine* (3) were specific to the professional responders.

The third important phase in the stories, often prompted by the interviewer, was the return from the mission. Three types of situations were described here: return to the family, return to work, and return to a *normal* life-a recurring theme, with a discussion of the boundaries between the normal and the pathological (e.g., in the context of psychological follow-up). As with the survivors, the most familiar points of reference seemed to have been blurred, and it was no longer easy to ascribe a normal, *taken for granted*, routine character to the ordinary situations of everyday life (professional and extraprofessional).

## Private space: the bereaved and survivors’ relatives

We were able to identify two, less publicized, secondary roles within the first circle of Study 1,000: the bereaved, and relatives of survivors. These two roles both belonged to the private space (i.e., personal relationships). They were played mainly by women (75%) in their 50s and over, such that bereaved mothers and mothers of survivors were over-represented in the sample.

The private space has undergone profound changes with the emergence of modern society (Giddens, 2006). As Luhmann (1980) showed, modernity is characterized by a *double expansion* of human relationships, with more opportunities for impersonal relationships, and more intense personal relationships. In this context, a specific language for personal relationships (that of love/friendship) was created in the fictional literature of the eighteenth century. In this lexical field (i.e., a symbolic universe), it is not permissible to exclude the personal aspect from communication. The function of this specific medium is to enable, cultivate, and promote the communicative processing of individuality, while at the same time differentiating between the near and distant worlds, between personal experience and the anonymously constituted world.

Unlike the professionals, who referred to victims as anonymous individuals (or bodies, injured, corpses, etc.), the bereaved referred to victims as individuals, calling them by their first names (Table 10). First names therefore had the highest specificity index in this category of testimonies. Next came the lexical forms designating family roles (and family space). Particular lexical fields were also used to describe families’ (very specific) mourning process, which involved autopsies, burials, and legal procedures. Several adjectives were used to describe either the deceased or the ceremony (*magnificent*, *beautiful*, and *kind*). Two specific lexical forms expressed the *pain* and *grief* of the bereaved. Again, it was the social role (member of a bereaved family) that structured stories and memories of the event.^16^

**Table 10:** The bereaved and survivors’ relatives: specificity indices.

Social role	Category	Lexical forms (specificity indices)
Bereaved (*n* = 42)	First name	Valentin, Caroline, Sébastien, Louise, Hugo, Camille, Stéphane, Hyacinthe, Sylvie, etc.
	Family role	Sister (68), mother (66), son (51), husband (50), parents (43), daughter (42), daddy (40), daughter-in-law (30), child (26), brother (25), father (20), grandson (19), ex-husband (17), cousin (16)
	Family space	Home (26)
	Funeral vocabulary	Burial (113), tribute (64), funeral (59), death (47), ceremony (46), photo (44), dying (30), burying (28), mourning (21), music (20), cemetery (20), Père-Lachaise (19), funeral (17), mass (16), bury (15)
	Forensic vocabulary	Laboratory (46), forensic (45), hospital (19), crutch (18), morgue (18), medico-legal (16), autopsy (16), body (14)
	Legal vocabulary (and victim support charity)	Lawyer (35), foundation (34), charity (32)
	Qualifier	Magnificent (30), beautiful (29), kind (19)
	Emotion	Pain (25), grief (17)
Relatives of survivor (*n* = 25)	First names	Paul-Romain, Jessica, Eva, Leo, Yohann, etc.
	Family roles	Daughter (64), wife (29), husband (25), father-in-law (22), son-in-law (22), sister (12)
	Role of a relative	Friend (14)
	Medical vocabulary	Hospital (33), amputate (17), amputation (16), Salpêtrière (16), transplant (12), psychologist (12), hosto (11)

For the relatives of survivors, the lexical fields were similar to those of the bereaved, although less pronounced and less varied, with the exception of specific medical terms.

## Public space: witnesses and local people

We were dealing here with two very distinct roles, based on the difference between direct and indirect witnesses: witnesses (at their window), who belonged to Circle 1, and local people (residents and users), who made up Circle 2. Both roles were played mainly by women (in their 30 and 40s), who made up 69% of witnesses and 73% of local residents.

The witnesses’ (Circle 1) lexical field was characterized above all by indicators of place: prepositions of place and lexical forms designating the observation post (*window*), public space, and location of the attacks. These were followed by other lexical forms used to describe what the witnesses saw, heard, and felt (Table 11).

**Table 11:** Witnesses/local people: specificity indices.

Social role	Category	Lexical forms (specificity indices)
Witnesses (*n* = 42)	Observation point	Window (129), building (76), neighbor (34), flat (30)
	Public space	Neighborhood (68), crossroads (43), boulevard (22), sidewalk (19)
	Location of attacks	Restaurant (52), Cambodge (38), Petit (35), Bière (28), Carillon (19), Comptoir (18)
	Prepositions of place	At (51), below (46)
	Visual perceptions	People (50), injured (14)
	Auditory perceptions	A kind of (44), noise (27), boom (20), hear (15)
	Emotions/feelings	Crying (18), unbelievable (14)
Local people (*n* = 144)	Neighborhood	District (173), street (130), dwelling (87), Charonne (39), 11th (29), République (24), Roquette (16), boulevard (14), Montreuil (14), neighbor (11)
	Prepositions/place markers	At (52), in front of (34), beside (16), place (14), location (12)
	Location of police operation on 18/11	Saint-Denis (125), Corbillon (21)
	Attack locations	Belle (75), Équipe (65), Cambodge (43), Petit (37), Carillon (32), café (18), restaurant (11)
	Consequences for neighborhood	Siren (77), helicopter (23), school (87), pupil (58), nursery (54), child (52), teacher (20), class (18), subway (35), bicycle (24), flower (31), candle (7)
	Emotions	Very (27), fear (26), hyper (16), bizarre (13), strange (12), fear (11), impression (10), freak out (8)

Circle 2 consisted of residents (*n* = 103) and *users* (*n* = 41; mainly shopkeepers and self-employed) of the neighborhoods affected by the attacks. Whereas residents tended to use the first-person singular *I* (21), users tended to use the impersonal or collective form *one* (14), albeit less frequently than *I*. Apart from this slight pronominal difference, these two categories did not differ significantly at the lexical level. For this reason, we treated residents and users as a single subcorpus (local people), in order to highlight their specific characteristics.

Local people play an important role, insofar as they represent an area’s living memory. The expression *I remember* was specific to this subcorpus (19). As with the Circle 1 witnesses, location was central to their testimonies, with prepositions and place markers, places in the neighborhood, the places of the attacks, and police operations.

Another specific feature of this subcorpus, which was directly related to the social role of the local resident or user, was a very detailed description of the consequences of the attacks on life in the neighborhood: noise of the sirens and helicopters, organization of local schools and day nurseries, presence of flowers and candles, and so on (Table 11). As *guardians* of their neighborhood’s memory (Truc, 2019), many complained about the sightseers who wandered around the district after the attacks, looking for traces (bullet holes, blood stains, etc.). A set of lexical forms specific to this subcorpus was used to express the emotional consequences for the district (e.g., *fear*, *anxiety*, *strange impressions*, and *hyper-weird atmosphere*).

Another specific feature of this corpus is the explicit link drawn between the attacks of November 13 and those of Charlie-Hebdo perpetrated 10 months earlier (*Charlie*: 29; often associated with *Hebdo*: 15), which referred directly to the neighborhood’s traumatic memory. The lower number of references to the Hypercacher supermarket and the police officer shot dead in Montrouge is interesting: the neighborhood’s uniqueness reinforces a phenomenon observed elsewhere in the general population (Hoibian et al., 2023).

## Remote spectators: representing public opinion?

The term *remote spectator* brings together the roles of the television viewer and the Internet and social media user. Both relate to the mass media, one of the functional systems of modern society. *Mass media* generally refers to all technical means of multiple reproduction for the dissemination of communication in society (Luhmann, 2012, 8). In modern society, as Luhmann notes, the mass media produce both transparent and nontransparent representations of public opinion. The main feature of the mass media system is that there is no interaction between sender and receiver: it is via a screen (television, computer, smartphone, etc.) that viewers access, experience and remember events. The use of technology precludes any direct interaction, which greatly increases the possibilities of communication.

As to how the mass media (in the broadest sense) influence the memory of remote viewers, from an empirical point of view, this is generally very difficult to address objectively in social science research, given the wide variety of broadcast media available today and the wide range of social uses that are difficult to compare. Nevertheless, in the present study, we had the data of individuals who could be objectively divided into those who were *present* (Circles 1 and 2) and those who were *absent* (Circles 3 and 4). These data therefore allowed us to compare the influence of the mass media on the construction of individual and collective memories, distinguishing between those who depended on the mass media to experience and remember the event (absent) and those who did not (present).

### Spectators at a distance: the view from the periphery (Circle 4)

Starting with the spectators who were furthest away from the epicenter of the attacks, Circle 4 interviewees produced the shortest testimonies (see Table 2). They exhibited a degree of unease, probably linked to the interview situation and the legitimacy of their testimony as (remote) spectators of the events (and moreover from other cities): *yes* (1,000), *no* (1,000), *maybe* (78), *rather* (50), *or* (44), *pessimistic* (39), *optimistic* (29).

**Figure 2:**
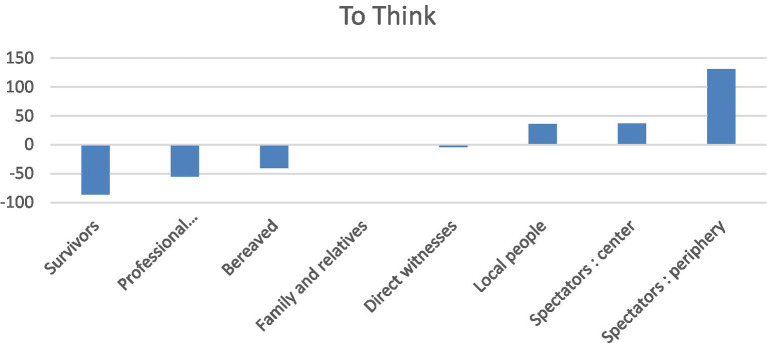
To think: specificity indices according to social role.

The narratives were quite analytical: *to think* (131), *true* (48), *analysis* (25), *precisely* (19), and *necessarily* (15). Participants tried to get *personally* involved (31). The verb *to think* was a lexical specificity emblematic of spectators on the periphery, indicating a certain level of reflexivity compared with the accounts of survivors or professional responders (closer to the facts and sensations): it had a lower specificity index closer to the center (Figure 2). Conversely, the terms *smell*, *blood* and *powder* had negative specificity indices, but these increased closer to the center.

Not surprisingly, the mass media were pivotal to the peripheral spectators’ stories (Table 12).

**Table 12:** Peripheral spectators (Circle 4): specificity indices.

Category	Lexical forms (specificity indices)
Omnipresence of mass media	Media (79), event (154), image (96), information (69), TV (67), television (43), radio (41), publicized (35), channel (29), news (22), BFMTV (21), loop (19), Internet (18), *Le Monde* (18), latest news (16), program (13), newspaper (13), press (12)
Official name of event	Attack (122)
Link to other similar events	Charlie (64), Hebdo (42)
Metaphor	War (62)
Religion and immigration policies	Religion (91), religious (16), Muslim (34), Algeria (27), Islam (25), conflation? (18), Arab (16), fundamentalism (14), Daesh (12), jihadists (12), North African (11)
Political power	Politics (13)
Globalization	Society (25), social (10), civilization (16), global (29), world (10)
Generations	Young people (49), student (47)
Key value	Freedom (27)
Emotions	Powerlessness (69), feeling (39), fear (29), incomprehension (22), mistrust (17), sadness (17), helplessness (16), anger (16), insecurity (14), scare (12), disgust (12), worry (10)

According to Luhmann, the societal function of the mass media lies not in the amount of updated information they provide, but in the *social memory that is produced*. The mass media produce a *latent everyday culture*, *background knowledge* that can serve as a starting point for communication:

Memory consists in the fact that, in every communication, certain statements about reality can be taken as known without having to introduce them into the communication and justify them. Memory is at work in all the operations of the system of society, that is, in all communications (Luhmann, 2012, 91–92).

The mass media guarantee all functional systems a present that extends to the whole of society and is known to all individuals (background knowledge).

The function of memory is to achieve a continuous discrimination between forgetting and remembering, which accompanies all observations of the system. The main performance here is in forgetting; it is only exceptionally that we remember something. Without the operation of forgetting, without the liberation for new operations, the system would have no future (Luhmann, 2012, 137).

The mass media perform this memory function for the system of society as a whole. As they form a functionally differentiated system, they have a universal competence for this specific function.

The operation of selecting what to remember and what to forget enables the social system to constantly say and re-say what is and what is not. This is the main role of *institutions* (De Munck, 1999; Boltanski, 2009) within social systems, and is reminiscent of the *re-entries* (loops or hypercycles) within the central nervous system (Edelman and Tononi, 2000). The mass media have become a globally important *generator of meaning*. In the case of the November 13 attacks, this function of semantic securitization required them to name and describe (*attacks*, *war*, *terrorism*, etc.) the event, and to provide *scripts* (Abelson, 1981) and interpretation schemas. This raises the question of what viewers retained.

Table 12 shows the main lexical forms specific to Circle 4. It shows the official name of the event (*attack*) adopted by the country’s politicians, its characterization (*war*) initiated by professional responders and the President of the Republic, the chronological and topographical link with Charlie Hebdo evoked by local people, and a set of forms that make it possible to envisage the multiple causes and meanings of this media event. Religion emerged as a central theme in the Circle 4 testimonies, closely linked to France’s immigration policies, its colonial past in North Africa (*Algeria*) and its foreign policy in the Middle East. In France, a secular state (as many stressed), religion and politics seemed inextricably linked in the interpretation of the event and its multiple and intertwined meanings in the context of a globalized society. The issue of generations was also raised (especially by the over-40s): the two terms most often used to designate the *targets* were *students* (attacks) and *young people* (French education and immigration policies).

Each of these (lexical) forms has two sides: one side that states and names, and the other that leaves in the shadows (*unmarked space*). Each of these forms allows for meanings to be formulated positively or negatively, accepted or rejected, and so on. In this respect, the use of the term *conflation* (18) seems to be another indicator of the reflexivity that characterized the Circle 4 testimonies. This term offered peripheral spectators the possibility of rejecting certain simplifications conveyed by the mass media. It should be noted that the mass media do not aim to produce a consensual *reality*. On the contrary, they produce an abundance of diverse opinions, and constantly work to irritate and discredit themselves (e.g., criticizing, discussing, challenging, and correcting themselves). The media propose forms (rather than imposing content) that allow people’s systems of consciousness to participate in the communication, by either accepting or rejecting it. As we saw earlier, they also provide scripts (arrangements of forms) that enable events to be followed.

Another function of the mass media is to irritate the social system, make it more sensitive to criticism (expecting surprises, news, something new, deviance, conflict, and irritation), and keep it alert and on the alert, constantly disturbing it like an immune system (Luhmann, 2012, 36). Irritability seems to be the most general structural characteristic of *autopoietic* systems (closely related to system memory), and the mass media can be viewed as a system of self-observation of society that constantly produces and enacts irritations (Luhmann, 2012, 132). For Luhmann, the main selectors of information are surprise, conflictuality, quantities, local reference, individual cases, and transgression of norms and values (here, *freedom*). According to these principles of selection, which govern society’s expectations in terms of what should be regarded as information and what, on the contrary, should remain valueless, the November 13 attacks in Paris were an exceptional media event. The mass media also irritate individual systems of consciousness, which use the language of emotions to express what it is expected of them as attentive spectators concerned by the news (Table 12).

### Difference between the center and the periphery: spectators in Circle 3

Two types of remote spectators were represented in our corpus: those belonging to the center (Paris and Ile-de-France), and those belonging to the periphery (Caen, Metz, Montpellier). This difference between the center and the periphery reflected a key characteristic of the French political system, with its centralized state in which the capital serves as the epicenter and sole point of reference for the rest of the country (periphery). The decentralization policies that have emerged since the 1980s have only served to confirm and reinforce this model of state centrality, insofar as they have always emanated from the center. The difference between the center and the periphery is so great in France that other functional subsystems constantly refer to it, not least the scientific system (for classifying journals and laboratories). In the case of the 13-November Program, this difference dictated the collection of data, as the sample was constructed in concentric circles. The perpetrators of the attacks also used this difference to increase the profile and symbolic power of their act. Traces of this can be seen in the testimonies of spectators from the other cities (Circle 4), where location markers (center/periphery) have high specificity indices: *Paris* (167), *country* (85), *France* (84),^17^ *French* (29), *region* (19), and, of course, *Metz* (152), *Montpellier* (152), and *Caen* (74), which were overused in each of the three city subcorpuses.

What were the differences between the narratives in Circle 3 (Paris) and Circle 4 (other cities)? How did memories of the center differ from those of the periphery? The first observation we made was that Circle 3 narratives (*n* = 147) were the least specific. In our opinion, this can be explained by the superimposition of (or confusion between) social roles present in the other three circles, starting with two roles that were characteristic of the families and relatives of survivors (Circle 1): *friend* (24) and *relative* (15). Some terms specific to local people emphasized the social role of the public transport user (Circle 2): *live in* (27), *Parisian* (18), *subway* (17), *transport* (15), and *RER* (9). There was also an explicit mention of *Charlie-Hebdo* (14).

Analysis revealed many similarities with Circle 4 spectators, such as markers of hesitation or discomfort: *yes* (50), *or* (27), and *no* (8). Above all, we found markers of a more analytical narrative (*idea*, *obviously*), fueled by social media (*Facebook*), mass media, and the causal scripts they disseminate (Table 13).

**Table 13:** Center and periphery: specificity indices.

Category	Lexical forms	Circle 3	Circle 4
Analytical narrative	To think	37	131
	Idea	20	0
	Obviously	13	1
	Necessarily	12	15
	Exactly	11	19
Mass media	Information	24	69
	Event	21	154
	Newspaper	13	13
	Facebook	13	1
	Channel	11	29
Description of the event	Attack	62	122
	Attack (verb)	24	1
	Kill	19	3
	War	19	62
	Terrorism	14	6
Interpretations and causes	France	49	84
	Country	36	85
	Politics	32	13
	Muslim	30	34
	Society	27	25
	French	13	29
	Racism	12	6
	Religion	11	91
	Daesh	10	12
	Arabic	10	16
	Koran	9	3
	Fanaticism	9	4
	Islamic	8	5
	Radicalization	7	0
Emotions	Touch	37	32
	Fear	21	29
	Sad	8	0
	Sadness	2	17
	Worry	7	10
	Anger	0	16
	Memorials	12	−1

In terms of characterizing the event, the term *terrorism* was an important feature, with a higher specificity index (14) in Circle 3 than in the rest of the corpus. The verbs *to attack* (24) and *to kill* (19) were also specific to spectators in Circle 3 (i.e., center). Even more so than in the other cities, politics (e.g., immigration policy and its effects on French society in terms of racism) seemed to influence how spectators in the Paris region (i.e., at the heart of the event and at the center of the political system) interpreted the attacks. Other concepts (*Koran*, *fanaticism*, *Islamic*, *radicalize*) that were highly specific to Circle 3 were used to raise the issue of religion. Memorials were also mentioned and were specific to Circle 3. Finally, the term *reaction* (22) could also be identified as being specific to Circle 3 for two main uses: describing an *initial reaction* to the events, and evoking the political reactions (mainly related to security) and their practical consequences for everyday life in Paris.

## Conclusion

The value of the present study is 2-fold. First, it provides an initial analysis of the entire corpus of Study 1,000 (Phase 1) of the transdisciplinary 13-November research Program. Parts of this corpus have already been processed (e.g., focusing on survivors, law enforcement officers, or certain remote spectators), but this is the first full analysis (together with the study by Lacoste et al., 2024). Second, it validates an original and stimulating research hypothesis for *memory studies* whereby social roles exert an important influence on the operations of individual systems of consciousness, particularly the operation of selecting what to remember and what to forget.

Based on a textometric analysis of 934 individual testimonies collected in 2016, we were able to explore collective memories of November 13 through the prism of these social roles. Each social role corresponded to a set of lexical forms through which people forgot and remembered November 13, 2015 (as a media spectacle, a professional intervention, a violent attack, a bereavement, a neighborhood taken by storm, etc.), and a specific vocabulary that gave this (remembered) event a particular meaning. The aim of the present study was in no way to discover new scientific truths about how to behave as a victim, a professional responder or a television viewer of an attack, but rather to show the importance of these social roles in the construction of individual and collective memories. Its contribution has less to do with the specific vocabulary of social roles, and more to do with the fact that these roles effectively influenced the lexical content of testimonies, and therefore constitute a particularly fertile entry point for exploring the collective memory of the Paris attacks in 2015.

We feel it is important to stress the specific nature of our study subject (i.e., testimony), in terms of its autonomy and reflexivity. The 934 individual testimonies were all entirely singular self-descriptions of *the same* event. In another words, the interviewees did not talk about November 13 in general, but about themselves through the prism of the role they played in this social tragedy. *Autology* (or self-reference) is an essential feature of complex systems (i.e., *self galaxy*).^18^ The analytical challenge was therefore to describe how our subject described itself and observe how it observed itself, in order to identify how its self-observations and self-descriptions were structured (Luhmann, 2021). How did interviewees reconstruct the past for future use? What differences did our interviewees’ individual systems of consciousness use (in the present) to remember and recount *their* November 13, when summoned to produce a testimony in a research laboratory? Analysis revealed that social role was the *difference that made all the difference*, in that it served as a filter for selecting what to remember and what to forget.^19^ The present research offers an original counterpoint to decontextualized studies of individual and collective. The issue of social role, a blind spot in *memory studies*, seems a worthwhile avenue to explore, applying a multidisciplinary and longitudinal approach. It would be interesting to observe how memory narratives change over time and whether they crystallize around templates or scripts specific to certain social roles. From a longitudinal perspective, we could also look at the different memory functions (Bluck and Alea, 2009) of these narratives, asking whether they enable survivors to integrate the event into their lives and give it meaning, professionals to better anticipate the future, and remote spectators to create social ties.

The forgetting is an important issue in the literature, but one that we can currently only study indirectly. If professional responders forgot the names and faces of victims, it was probably because their professional role protected them from this, directed their attention to other more relevant elements and created memories from this sorting (relevant/irrelevant). If the bereaved had little to say about the political dimension of the event, it was precisely because the grief they were experiencing took up all the space in their narrative. The same was true for survivors: factual details and perceptions of this singular event overshadowed everything else. There is nothing more to be said on this at this stage: only a longitudinal approach could confirm what each social role allows to be forgotten. This is one of the current limitations of the study and paves the way for future analyses.

Another fruitful avenue is undoubtedly that of gender: in our view, it would provide a means of studying the infinite psychological nuances in the interpretation of roles. Although the sample was very unevenly distributed according to gender, and we did not set out to deal specifically with this issue, certain trends are already clear from Phase 1 of Study 1,000. Across the whole corpus, women tended to talk more about their emotions than men, whether they were survivors, professional responders, or television viewers. They also talked a great deal about their loved ones, and seemed to care more about others (spouse, child, friend, etc.). For example, among Circle 3 (center) spectators, women (i.e., 65% of this subcorpus) talked more than men about family roles, the private space, and their emotions. While this was a general tendency among female spectators in Circles 3 and 4, it was more pronounced within Circle 3, owing to the geographical proximity to the event. Even more so than those living in other cities, women living in Paris worried about their children and relatives. As for the men, they referred to the *event* in terms of *attack* and *terrorism*. Their testimonies spoke of *debate*, *situation*, *problem*, and *conflict*. The terms *Arab* and *Muslim* also occurred more frequently. In Circle 4 (spectators from other cities), there was also a marked gender difference between the private space and the space of comment and opinion. Women, who made up 61% of this subcorpus, talked more about family, education, and their feelings. Men talked a lot about the football match at the Stade de France that they had been watching on television that evening. They also made particular use of the words *event*, *today*, and *society* to talk about November 13. Specific lexical forms reflected the more analytical nature of their description (*naturally*, *especially*, *reason*, *element*, *precise*, etc.). The interpretation of the peripheral spectators’ social role therefore varied not only according to their (geographical) distance from the event, as we have shown, but also according to their gender. An initial study carried out among interviewees living in Metz, using another TXM tool (factorial correspondence analysis), had already highlighted the importance of this key variable (Peschanski et al., 2023). Although this observation can be extended to all interviewees in Circles 3 and 4, it requires more detailed analysis and further development.

## Data availability statement

The original contributions presented in the study are included in the article/Supplementary material, further inquiries can be directed to the corresponding author.

## Ethics statement

The studies involving humans were approved by Agence Nationale de la Recherche, France. The studies were conducted in accordance with the local legislation and institutional requirements. The participants provided their written informed consent to participate in this study.

## Author contributions

J-FO: Writing – original draft, Writing – review & editing. SH: Software, Writing – review & editing. CK-P: Data curation, Project administration, Writing – review & editing. FE: Supervision, Writing – review & editing. DP: Supervision, Writing – review & editing.

## Footnotes

^1^Among the main conditions for the autonomy of social systems, Parsons notes in particular the existence of a catalog of roles that are sufficiently differentiated for individuals to be able to find a response to their needs throughout their lives without having to *leave society*.

^2^For example, in the sociology of work (Hughes, 1996) and occupations (Freidson, 1984), within formal and informal organizations (Goffman, 1968, 1973; Humphreys, 2007).

^3^It should be noted that a fourth collection phase is planned for 2026 (i.e., 10 years after the first one). The dropout rate for each new phase has so far been around 22%.

^4^This study proposed a 10-year protocol with four collection phases (1 week, 11 months, 35 months, and 119 months after the event). Proximity/distance to the event was a criterion for selecting participants: they came from different locations in the United States (New York, Boston, New Haven, Washington, St Louis, Palo Alto, Santa Cruz). Another selection criterion was to distinguish direct witnesses of the event from remote spectators (all those who learned of it by word of mouth, or through the media, or by telephone or email). A total of 206 individuals participated in the four phases of the study.

^5^Corpus ref.: TXM NOV13-P1-V2-2022-10-11 (version TXM-0.8.1 on Mac). To define the social roles, we use the *testimony-category* variable.

^6^It is important to underline the time needed for the tedious work of transcribing the testimonies (speech to text), correcting and preparing the data for textometric analysis. Phase 2 will soon be available for a longitudinal study.

^7^For example, a positive specificity index of 9 means that the word had less than one chance in 1 billion (1 followed by 9 zeros) of appearing with such a high frequency in the section under consideration. Mathematically, the index corresponds to the absolute value of the logarithm to base 10 of the probability of the word appearing with its frequency or more in the section (i.e., 10^−9^).

^8^However, the interviewers were given strict instructions never to intervene to correct a mistake.

^9^It should be noted that some studies on flashbulb memories extend the spectrum of analysis to include people who have directly experienced the event (see Er, 2003). This would be an interesting line of analysis to develop further.

^10^These statistical indices are shown in brackets after each word. We rounded down the indices provided by the TXM software to the nearest whole number, as the size of the corpus meant that there could be very high values, so the accuracy provided by the decimal point was not useful here. It should be noted that for all the observations mentioned here, we calculated the specificity of the *lemmas* (i.e., regardless of the words’ inflection: all verb forms reported in the infinitive, no singular/plural distinction, etc.).

^11^In TXM, 1,000 is the conventional value given when the specificity index is so high that it can no longer be calculated: the corresponding probability cannot be represented because it is so low, and too many zeros would be needed after the decimal point before the first non-zero digit (*p* = 0.000000000000). In other words, the singularity of these two lexical forms within the subcorpus was such that the probability of them having the same frequency of occurrence had they been distributed randomly was almost zero. There was therefore nothing random about the strength of their presence.

^12^10 at Carillon, 5 at Belle Equipe, 1 at Petit Cambodge, and 1 at Bonne Bière.

^13^Original tables (in French) are available in Supplementary material.

^14^*Powder* and *blood* were the two most frequent co-occurrents of *smell*, with specificity indices above 100.

^15^The co-occurrents of *blood* underscored this idea of copiousness: *everywhere*, *covered*, *pool*, *puddle*, and *full*.

^16^It should be noted that in order to maintain confidentiality, these are not their real first names. For personalities who could be recognized from their functions, we obviously followed a different protocol.

^17^It should be noted that the noun *France* took on several meanings here: country (different modalities: in France, France, from France, a France), location of attack (Stade de France), and media outlet (France 2, France Inter, France Info, France Culture, etc.).

^18^See, for example, Varela (2017).

^19^It should be noted that the factorial correspondence analysis of the 934 interviews carried out by Lacoste et al. (2024) confirmed this important finding.
