# Supplementary material for: Collective memories and social roles: the case of the Paris terrorist attacks of 13 November 2015
Source: Front Sociol. 2024 May 22;9:1388380. doi: 10.3389/fsoc.2024.1388380 (PMC11151273; doi:10.3389/fsoc.2024.1388380)
Supplement: Supplementary file 1 [file Data_Sheet_1.PDF]

Supplementary Material

Tables

Table 1. Étude 1000 (Phase 1) : répartition des témoignages par cercles (N=934)

Cercles	N	Catégorie	% femmes	% 18-39 ans
Cercle 1. Les attentats	359	survivants (112) / intervenants professionnels (138) / endeuillés (42) / témoins (42) / familles & proches (25)	49%	57%
Cercle 2. Les quartiers touchés	144	habitants (103) / usagers (41)	73%	59%
Cercle 3. L'Ile-de-France	147		65%	61%
Cercle 4. La Province	283	Caen (117) / Metz (76) / Montpellier (84) / Autre (6)	61%	42%
Non codé	*1*		*0%*	*100%*
**Total**	**934**		**59%**	**53%**

Table 2. Étude 1000 (Phase 1) : catégories retenues pour l'analyse

Rôles sociaux	Cercle	Nombre de témoignages	Longueur des sous-corpus (en mots)	Mots/témoin
Survivants	1	112	2.663.835	23.784
Intervenants professionnels	1	138	2.594.003	18.797
Endeuillés	1	42	1.025.903	24.426
Familles/proches des survivants	1	25	529.167	21.167
Témoins directs	1	42	887.568	21.133
Gens du quartier	2	144	2.276.621	15.810
Spectateurs à distance : centre	3	147	2.186.649	14.875
Spectateurs à distance : périphérie	4	283	1.735.278	6.132

Table 3. Spectateur/victime/survivant : indices de spécificité

Rôle social	Catégorie	Formes lexicales (indice de spécificité)
1. Spectateur au Bataclan	Événement musical	concert (224) ; Eagles (51) ; Death (48) ; Metal (42) ; groupe (41) ; chanson (20) ; rock (19) ; batterie (17) ; musique (14)
	Salle du Bataclan	fosse (174) ; salle (108) ; porte (66) ; sortie (64) ; scène (61) ; escalier (60) ; balcon (50) ; bar (36) ; toilette (31) ; loge (30) ; plafond (20)
2. Victime	Attaques	tirer (146) ; mec (143) ; balle (140) ; tir (77) ; terre (65) ; sol (64) ; blesser (49) ; recharger (34) ; trois (19) ; poudre (16) ; tireur (15)
	Verbes d'action	sortir (121) ; courir (61) ; allonger (53) ; crier (52) ; marcher (43) ; tomber (38) ; mourir (36) ; ramper (36) ; aider (31) ; plaindre (30) ; relever (30) ; attendre (28) ; hurler (25) ; respirer (26) ; gueuler (17) ; calmer (17) ; stresser (17) ; piétiner (14)
	Perceptions	bruit (65) ; pétard (50) ; lumière (41) ; odeur (40) ; cri (31)
	Parties du corps	sang (67) ; bras (56) ; jambe (55) ; main (50) ; tête (42) ; cuisse (22) ; épaule (16)
	Vêtements	veste (20) ; T-shirt (20)
3. Survivant	Dénomination du rôle	vivant (35) ; rescapé (34) ; survie (20) ; chance (14)
	Vocabulaire médical	psy (55) ; hôpital (44) ; psychiatre (32) ; thérapie (31)
	Vocabulaire juridique	déposition (70) ; avocat (29) ; plainte (18)
	Moyens de transport/communication	taxi (29) ; téléphone (24)

Table 4. Le rôle de spectateur au concert du Bataclan : extraits de témoignages

Et puis, au bout d'un moment, effectivement, j'entends comme des bruits de pétards... quelque chose de... à ce stade... j'avais donc la table de mixage juste derrière moi, je suis musicien: ça ressemble comme à un bruit de câble qu'on détache et à un truc qui pétarade dans les enceintes. Je me retourne vers la table de mixage, j'essaye de regarder et, en même temps, je me dis: "c'est absurde! Le son il vient de devant, il ne vient pas de derrière! Si ça pétaradait dans la table, ça pétaraderait devant et non pas derrière! Donc c'est autre chose!". (*PAR0147-Bataclan*)
Quand il y a des détonations qui ont explosé, enfin qui ont pété, et on n'a pas compris ce que c'était. Parce qu'on était en train de sauter, danser, c'était la joie, enfin, c'était vraiment un moment de pur joie, donc de de rebasculer à l'opposé vraiment c'était... c'était, c'était compliqué. Et j'ai mis beaucoup de temps à m'apercevoir ce qui s'était passé et et de le réaliser vraiment consciemment, me dire: "Il se passe... il y a des gens qui tirent avec des armes en fait.". J'essayais toujours de trouver des des des extrapolations des... "C'est un mouvement de panique, c'est des pétards." (…) Et j'ai, j'avais passé la tête par-dessus pour voir ce qui s'est passait mais... J'ai vu, j'ai vu un homme debout au fond. Et les spectateurs qui étaient allongés dans la fosse, j'ai pas vu de sang, j'ai pas... Et mon cerveau qui a refusé de de voir ce que c'était, je me suis dit :"Oh c'est beau, on dirait des blés couchés.". Mais je voyais pas, j'ai pas vu d'armes, je pense que, enfin je l'ai vu debout au fond de la fosse, je pense que... il l'avait. Que je l'ai vu, mais je l'ai pas, je l'ai pas enregistré. Et... donc on est parti en rampant le long du balcon et... on s'est relevé pour pour franchir la la porte, on s'est retrouvé dans une cage d'escalier. (*PAR0331-Bataclan*)
Et la première chose que j'ai entendu donc des bruits, des bruits assez assez, assez nets et intenses. Excusez-moi, j'ai eu deux premiers réflexes: le premier c'est de me dire bêtement: "le batteur a fait un pain". Parce que le bruit était il couvrait, enfin j'ai vite compris que ça couvrait le bruit de la caisse claire de la batterie, mais en tant que musicien le c'est le premier réflexe que j'ai eu, c'est de me dire, c'est bizarre il fait un pain alors qu'il joue super bien, il y a quelque chose qui va pas, j'entends des bruits de de de caisse claire, mais qui sont pas pas en rythme, et qui sont beaucoup trop forts. Et puis le second, la seconde pensée que j'ai eu, mais c'était immédiat ça s'est fait en une fraction de seconde, la seconde pensée que j'ai eu c'est: "la sono a dû a dû lâcher et ça fait ça fait ce bruit-là dans la sono" et je me retourne vers l'origine du bruit, donc vers l'entrée de la salle et puis, et puis ça continue, ça continue et je commence à entendre des gens qui qui qui crient et qui disent c'est des pétards c'est pétards c'est une blague, c'est une mauvaise blague etc. Et et bêtement, dans les premiers instants, quand j'ai supposé que c'était effectivement une mauvaise plaisanterie, et que j'ai commencé à entendre des gens, des gens hurler des, des, des, des choses en arabe, je me suis dit bon, c'est vraiment la la pire des blagues qu'on puisse faire dans un concert, c'est c'est vraiment complètement stupide. (…) C'est le fait d'avoir vu le batteur partir qui m'a fait comprendre que c'était pas un problème de batterie et que c'était pas un problème de sono donc voilà, là encore, tout ça s'est mélangé, très très vite dans ma tête. (*PAR0274 - Bataclan*)

Table 5. Le rôle de victime : extraits de témoignages

Comme si, pour ne pas mourir il suffisait de ne, de le vouloir. Je, je me fais un espèce d'auto-coaching, comme ça: en le voulant très fort, en tenant le coup... voilà. Donc, je me concentre sur le fait de ne pas bouger, de respirer sans que mon dos... sans que ça se voit. Et puis, je me dis que si je survis, il faudra que je témoigne. Je suis journaliste, donc je commence à rentrer dans une posture de reporter de guerre, ce qui n'est pas du tout mon métier par ailleurs parce que moi je bosse sur le cinéma, donc vraiment, rien à voir. Je suis pas du tout taillé pour ce genre de... mais bon. Je me, je me dis qu'il faut que j'analyse tout ce qui se passe, que j'enregistre le moindre son, les moindres gestes, les moindres vibrations. Et le parquet c'est du bois, ça transmet le son, je me... c'est comme si j'étais un Sioux qui est avec l'oreille collée contre le le parquet. J'essaie de voir s'il y a des déambulations, s'il y a des gens qui sont toujours vivants autour de nous qui bougent ou pas ou... enfin, j'essaie de comprendre ce qui, qui se passe. (…) Moi, à ce stade, je me raccroche à plein de trucs, parce qu'il faut se... parce que, pour survivre, il faut... il y a deux choses. La première, c'est un truc qui... quelque part fait vriller le cerveau, mais pour, pour avoir une chance de survivre, il fallait rester calme, pour rester calme, il fallait accepter sereinement de mourir. C'est une contradiction folle mais... mais j'ai fini par me dire ça. Donc j'ai, à un moment donné, il a fallu que j'envisage que mon fils serait bel et bien orphelin. (*PAR0147-Bataclan*)
J'ai eu ce moment où j'ai lâché prise totalement. (…) Il y a eu ce moment où j'ai totalement lâché prise et je me rappelle... Je me rappelle avoir regardé Thomas, en me disant: "Tu sortiras pas de cette salle. J'étais résigné. Et puis euh... Il y a eu... Juste après ça un moment de de de prise de conscience, l'instinct de survie peut-être. Et j'aime beaucoup les jeux vidéo. Je sais que ça peut paraître hors propos là comme ça tout de suite, mais vous allez voir, ça a du sens. Parce que j'ai travaillé, j'ai travaillé dix ans pour une une petite chaîne télé dans laquelle je j'étais journaliste et je parlais de jeux vidéo, du matin au soir. Et ce moment où l'instinct de survie a repris le dessus, quand j'ai relevé la tête. J'étais... J'ai analysé la la situation comme je l'aurais analysée dans un jeu de de de guerre, dans un jeu de, un Call of duty, un Battlefield ou peu importe. C'est complètement, complètement absurde, ce que je suis en train de dire, mais sur le moment, j'ai relevé la tête et je me suis dit: "Voilà essaye d'analyser la situation, essaye de de comprendre vraiment ce qui est en train de se passer. Regarde où tu peux sortir, regarde qui fait quoi et regarde qui a quoi comme arme dans les mains". Et donc j'ai vu que le gars de gauche qui qui était habillé en noir, était en train de recharger son arme. Et il avait le dos tourné. Et j'ai vu que celui de droite, était en train de hurler sur des gens, mais il avait aussi le dos tourné. Ca s'est joué vraiment sur une fraction de seconde. J'ai regardé Thomas. J'étais pétrifié, j'étais vraiment je, je, je, j'arrivais plus à sortir un son, mais je l'ai regardé, je lui ai fait un signe de tête pour lui indiquer la la la porte et lui faire comprendre que j'allais me lever. (…) Donc je l'ai regardé, j'ai fait ce signe de tête, et j'ai regardé le jeune homme qui était, qui était allongé sur sur sur mes jambes. J'ai tiré un peu sur mes jambes, il a compris que je voulais me lever. Il s'est dégagé légèrement sur le côté. Je me suis levé, j'ai perdu une chaussure. Et je suis parti, en courant. (*PAR0274-Bataclan*)

Table 6. Le rôle de survivant : extraits de témoignages

Mais, mais j'ai mis du temps à me à me considérer comme victime ou dommage collatéral de des événements. Et puis, même encore aujourd'hui, de temps en temps, je me pose un peu la question de me dire: "Mais... où est le trauma?" Mais voilà, le fait de faire ce ce mouvement-là, je je me suis bien rendu... enfin à ce moment-là, je j'ai j'ai un peu accepté l'idée que, malgré tout, malgré tout, j'étais victime victime indirecte des événements, mais victime parce que, parce que j'avais besoin de me faire violence pour revenir là, parce que... parce que ça a créé des larmes, parce que... parce que je le gérais pas du tout quoi. (*PAR0107*)
Mais une victime du 13 novembre, voilà, physiquement il m'est rien arrivé, physiquement. Donc je me sens pas victime quand quand je vois tout ce qui s'est passé autour et et tous les blessés qu'il y a eu et toutes les vies qui ont été chamboulées, je me je me sens, enfin je j'estime pas être sur la même échelle, en fait, de de gravité que ces personnes, donc je peux pas me considérer victime. (*PAR0332*)

Table 7. Les intervenants professionnels (N=138) : catégories et effectifs

Catégories	Rôle professionnel (N)
Forces de l'ordre<br>(N=61)	Policier/Fonctionnaire de police/gardien de la paix/RAID/BRI : 33 ; Police technique (enquêteur) : 3 ; Maître-chien (explosif) : 2 ; Démineur : 2 ; Chauffeur (police) : 1 ;<br>Chef de brigade/équipe/service : 3 ; Commandant de police : 2 ; Major de police : 1 ; Directeur de police : 1 ; Chef de la BRI : 1 ; Officier BRI : 1 ; Commissaire de police : 11
Professionnels de la santé (N=21)	Infirmier : 5 ; Médecin généraliste : 1 ; Médecin urgentiste : 2 ; Médecin BRI :1 ; Étudiant en médecine : 1 ; Assistant médical : 1 ; Chef de service hôpital : 1 ;<br>Psychologue : 6 ; Psychiatre : 3
Secouristes<br>(N=21)	Croix-Rouge : 9 ; Secouriste (protection civile) : 3 ; Pompier : 9
Acteurs politiques et agents administratifs<br>(N=24)	Élu ou employé municipal (Paris ; 11è ; 10è) : 17 ; Député : 2 ; Cabinet de l'Élysée : 2 ; Président de la République ; Secrétaire d'État à la défense (Anciens combattants) ; Ministre de l'intérieur
Autres<br>(N=11)	Juriste/Avocat (aide/prise en charge des victimes) : 3 ; Service propreté de la ville : 2 ; Journaliste : 2 ; Agents de sécurité (stade de France) : 2 ; Directeur d'école : 1

Table 8. Le langage des professions comme univers de discours : indices de spécificité

**Catégories**	**Formes lexicales (indice de spécificité)**
Services en présence	BRI (245) ; police (203) ; RAID (116) ; pompier (128) ; démineur (108) ; Croix-Rouge (93) ; secouriste (90) ; policier (78) ; judiciaire (77) ; chien (76) ; secours (76) ; CUMP (68) ; SAMU (64) ; psychologue (52) ; médical (49) ; déminage (45) ; médecin (44) ; intervenant (41)
Grades	chef (225) ; commissaire (155) ; agent (98) ; officier (97) ; directeur (79) ; adjoint (54) ; équipier (36) ; gradé (35) ; enquêteur (35) ; capitaine (35) ; major (31)
Collectif de travail	colonne (222) ; équipe (211) ; effectif (159) ; caserne (128) ; brigade (100) ; unité (100) ; commissariat (98) ; opérationnel (centre ou soutien 94) ; équipage (92) ; renfort (62) ; cellule (de crise ou d'urgence 58) ; urgence (53) ; zone (51)
Ordres	mission (146) ; charge (130) ; assaut (120) ; ondes (radios 114) ; débriefing (92) ; dispositif (87) ; périmètre (79) ; commandement (65) ; hiérarchie (55) ; état-major (47) ; direction (46) ; sécurisation (45) ; progression (42) ; procédure (39)
Autorités politiques (et administratives)	fonctionnaire (145) ; mairie (144,5) ; maire (134) ; arrondissement (115) ; préfet (99) ; président (67) ; ministre (65) ; préfecture (52) ; autorité (39)
Outils de travail	gilet (127) ; matériel (123) ; engin (114) ; bouclier (88) ; équipement (75) ; arme (74) ; explosif (69) ; protection (47) ; armement (33) ; pare-balles (32) ; cartouche (27) ; casque (27) ; munition (22) ; grenade (21)
Victimes	personne (60) ; corps (50) ; blessé (47) ; blessure (31) ; cadavre (29).
Verbes d'action	intervenir (174) ; gérer (168) ; équiper (107) ; évacuer (88) ; progresser (74) ; sécuriser (72) ; former (54) ; organiser (53) ; préparer (43) ; neutraliser (43) ; riposter (20) ; diriger (20).
Cibles	terroriste (59) ; kamikaze (33)
Lieu d'intervention	Bataclan (35) ; Stade (35).

Table 9. Le rôle d'intervenant professionnel : extrait d'un témoignage

Mais c'est vrai que j'étais, j'étais heureux d'avoir une formation militaire avant de rentrer dans la police parce qu'on n'est pas du tout formé à intervenir sur ce genre de situation. Là, en l'occurrence, c'était vraiment une scène de guerre en plein Paris. Quand j'ai évacué les victimes, c'est l'évacuation de blessés par balle qui m'a servi, que j'avais apprise, que j'ai apprise dans l'armée. Quand je me mettais à couvert, je faisais des ouvertures d'angle que je progressais, je suis vraiment passé en mode automatique, j'ai fait ce que j'ai appris en tant que militaire. (*PAR0752*)

Sur le coup... Je me suis dit bah pour moi, pour bien faire comprendre aux gens ce que ça pouvait être pour moi, ça, ça aurait pu être une scène de guerre. Pour comprendre un peu, pour essayer de faire comprendre, c'est-à-dire un quartier où on entend des appels au secours, partout où il y a du sang un peu partout... voilà. Être aux aguets tout le temps, savoir s'il va pas y voir quelqu'un, si... Voilà, une scène de guerre. (*PAR0756*)

Quand je discute avec mes collègues du Raid, ils me disent en fait c'est une scène... C'est des scènes qu'ils ont l'habitude de voir à Kaboul, il dit pas en plein Paris en 2015. C'est... on est dans un contexte de guerre. (*PAR0807*)

Et nous, nous sommes descendus, nous sommes arrivés à l'angle, donc, du café La Bonne Bière, qui se trouve Fontaine-au-Roi, Faubourg du Temple. Et là, nous avons vu les premières victimes, donc des personnes allongées au sol, qui... qui avaient des blessures horribles, hein! Des blessures de guerre, c'est le mot qui est revenu très souvent de la part de tout le monde... après... je veux dire, après un débriefing. (*PAR0741*)

On s'est dirigé en direction de l'entrée du Bataclan et on a commencé à évacuer un maximum de victimes qui se trouvaient dans le hall d'entrée, qui menait à la Fosse. C'était... Disons qu'à cet endroit-là, la scène était assez... C'était une scène de guerre à l'entrée du Bataclan, il y avait du sang de partout, il y avait des, c'était du sang mêlé à des morceaux de vitres brisées. Il y a des collègues qui, en évacuant les victimes tombaient carrément dans la flaque de sang tellement c'était recouvert. (*PAR0752*)

Table 10. « Guerre » : indice de spécificité selon le rôle social

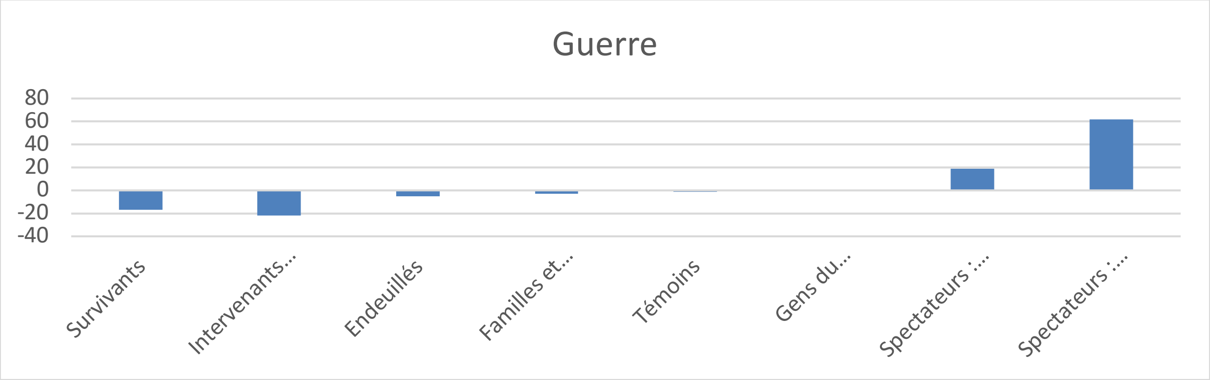

Table 11. Endeuillés / Familles et proches des survivants : indices de spécificité

Rôle social	Catégorie	Formes lexicales (indice de spécificité)
Endeuillés (N=42)	Prénoms	Valentin, Caroline, Sébastien, Louise, Hugo, Camille, Stéphane Hyacinthe, Sylvie, etc.
	Rôles familiaux	sœur (68), maman (66) ; fils (51) ; mari (50) ; parents (43) ; fille (42) ; papa (40) ; belle-fille (30) ; enfant (26) ; frère (25) ; père (20) ; petit-fils (19) ; ex-mari (17) ; cousin (16)
	Espace familial	maison (26)
	Vocabulaire funéraire	enterrement (113) ; hommage (64) ; obsèques (59) ; décès (47) ; cérémonie (46) ; photo (44) ; mourir (30) ; enterrer (28) ; deuil (21) ; musique (20) ; cimetière (20) ; Père-Lachaise (19) ; funèbre (17) ; messe (16) ; inhumer (15)
	Vocabulaire médico-légal	institut (46) ; médico-légal (45) ; hôpital (19) ; béquille (18) ; morgue (18) ; médicolégal (16) ; autopsie (16) ; corps (14)
	Vocabulaire juridique (et associatif d'aide aux victimes)	avocat (35) ; fondation (34) ; association (32)
	Qualificatif	magnifique (30) ; beau (29) ; gentil (19)
	Émotion	douleur (25) ; chagrin (17)
Familles et proches des survivants (N=25)	Prénoms	Paul-Romain, Jessica, Eva, Leo, Yohann, etc.
	Rôles familiaux	fille (64) ; femme (29) ; mari (25) ; beau-père (22) ; gendre (22) ; sœur (12)
	Rôle de « proche »	ami (14)
	Vocabulaire médical	hôpital (33) ; amputer (17) ; amputation (16) ; salpêtrière (16) ; greffe (12) ; psy (12) ; hosto (11)

Table 12. Témoins / Gens du quartier : indices de spécificité

Rôle social	Catégorie	Formes lexicales (indice de spécificité)
Témoins (N=42)	Poste d'observation	fenêtre (129) ; immeuble (76) ; voisin (34) ; appartement (30)
	Espace public	quartier (68) ; carrefour (43) ; boulevard (22) ; trottoir (19)
	Lieux des attaques	restaurant (52) ; Cambodge (38) ; Petit (35) ; Bière (28) ; Carillon (19) ; Comptoir (18)
	Prépositions de lieu	chez (51) ; bas (46)
	Perceptions visuelles	gens (50) ; blessés (14)
	Perceptions auditives	espèce (44) ; bruit (27) ; boum (20) ; entendre (15)
	Émotions / Ressenti	pleurer (18) ; incroyable (14)
Gens du quartier (N=144)	Le quartier	quartier (173) ; rue (130) ; habiter (87) ; Charonne (39) ; 11è (29) ; République (24) ; Roquette (16) ; boulevard (14) ; Montreuil (14) ; voisin (11)
	Prépositions/marqueurs de lieu	chez (52) ; devant (34) ; côté (16) ; lieu (14) ; endroit (12)
	Lieu de l'opération policière du 18/11	Saint-Denis (125) ; Corbillon (21)
	Lieux des attaques	Belle (75) ; Équipe (65) ; Cambodge (43) ; Petit (37) ; Carillon (32) ; café (18) ; restaurant (11)
	Conséquences sur le quartier	sirène (77) ; hélicoptère (23)<br>école (87) ; élève (58) ; crèche (54) ; enfant (52) ; enseignant (20) ; classe (18)<br>métro (35) ; vélo (24)<br>fleur (31) ; bougie (7)
	Émotions	très (27) ; peur (26) ; hyper (16) ; bizarre (13) ; étrange (12) ; angoisse (11) ; impression (10) ; flipper (8)

Table 13. « Penser » : indice de spécificité selon le rôle social

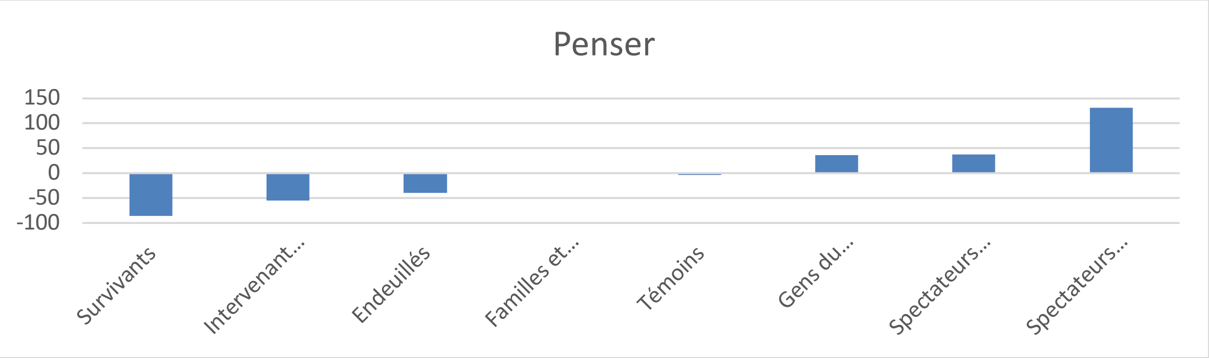

Table 14. Les spectateurs de province (cercle 4) : indices de spécificité

Catégories	Formes lexicales (indice de spécificité)
Omniprésence des médias de masse	médias (79) ; événement (154) ; image (96) ; information (69) ; télé (67) ; télévision (43) ; radio (41) ; médiatique (35) ; chaîne (29) ; infos (22) ; BFMTV (21) ; boucle (19) ; internet (18) ; Le Monde (18) ; actualité (16) ; émission (13) ; journal (13) ; presse (12).
Dénomination officielle de l'événement	attentat (122)
Lien avec d'autres événements similaires	Charlie (64) ; Hebdo (42)
Métaphore	guerre (62)
Religion et politiques d'immigration	religion (91) ; religieux (16) ; musulman (34) ; Algérie (27) ; Islam (25) ; amalgame (18) ; arabe (16) ; intégrisme (14) ; Daech (12) ; djihadistes (12) ; maghrébin (11)
Pouvoir politique	politique (13)
Mondialisation de la société	société (25) ; social (10) ; civilisation (16) ; mondial (29) ; monde (10)
Générations	jeune (49) ; étudiant (47)
Valeur-clé	liberté (27)
Émotions	impuissance (69) ; sentiment (39) **;** peur (29) ; incompréhension (22) ; méfiance (17) ; tristesse (17) ; impuissant (16) ; colère (16) **;** insécurité (14) ; crainte (12) ; dégoût (12) ; inquiétude (10)

Table 15. Centre et périphérie : indices de spécificité

**Catégorie**	**Formes lexicales**	**C-3**	**C-4**
Récit analytique	penser	37	131
	idée	20	0
	évidemment	13	1
	forcément	12	15
	justement	11	19
Médias de masse	information	24	69
	événement	21	154
	journal	13	13
	facebook	13	1
	chaîne	11	29
Qualification de l'événement	attentat	62	122
	attaquer	24	1
	tuer	19	3
	guerre	19	62
	terrorisme	14	6
Interprétations et causes	France	49	84
	pays	36	85
	politique	32	13
	musulman	30	34
	société	27	25
	français	13	29
	racisme	12	6
	religion	11	91

	Daech	10	12
	arabe	10	16
	coran	9	3
	fanatisme	9	4
	islamique	8	5
	radicaliser	7	0
Émotions	toucher	37	32
	peur	21	29
	triste	8	0
	tristesse	2	17
	inquiétude	7	10
	colère	0	16
	Mémoriaux	12	-1
